# Screening of mRNA markers in early bovine tuberculosis blood samples

**DOI:** 10.3389/fvets.2024.1330693

**Published:** 2024-04-05

**Authors:** Dongfeng Jiang, Xiaoyi Song, Liyu Yang, Li Zheng, Kaifeng Niu, Hui Niu

**Affiliations:** ^1^College of Animal Science and Technology, Henan University of Animal Husbandry and Economics, Zhengzhou, China; ^2^Henan Province Animal Reproductive Control Engineering Technology Research Center, Zhengzhou, China

**Keywords:** bovine tuberculosis, RNA-Seq, weighted gene co-expression network, immune regulation, disease diagnostic model

## Abstract

Bovine tuberculosis (bTB) is a chronic zoonotic disease caused by *Mycobacterium bovis*. A large number of cattle are infected with bTB every year, resulting in huge economic losses. How to control bTB is an important issue in the current global livestock economy. In this study, the original transcriptome sequences related to this study were obtained from the dataset GSE192537 by searching the Gene Expression Omnibus (GEO) database. Our differential gene analysis showed that there were obvious biological activities related to immune activation and immune regulation in the early stage of bTB. Immune-related biological processes were more active in the early stage of bTB than in the late. There were obvious immune activation and immune cell recruitment in the early stage of bTB. Regulations in immune receptors are associated with pathophysiological processes of the early stage of bTB. A gene module consisting of 236 genes significantly related to the early stage of bTB was obtained by weighted gene co-expression network analysis, and 18 hub genes were further identified as potential biomarkers or therapeutic targets. Finally, by random forest algorithm and logistic regression modeling, FCRL1 was identified as a representative mRNA marker in early bTB blood. FCRL1 has the potential to be a diagnostic biomarker in early bTB.

## 1 Introduction

Bovine tuberculosis (bTB) is a chronic zoonotic disease caused by *Mycobacterium bovis* (1). As a member of the *M. tuberculosis* complex (MTBC), *Mycobacterium bovis* is 99.9% similar to *M. tuberculosis* humans at the genomic level (2). A comprehensive econometric analysis combining agricultural production and human health indicators shows that bTB is currently one of the most serious livestock diseases in the world (3). According to statistics, about 50 million cattle worldwide are infected with *M. bovis* each year, and the associated economic losses can be as high as $3 billion (4). For the current global livestock economy, how to better control bTB is still an important issue (5). The disease was also defined as a Category B Animal disease by the World Organization for Animal Health (OIE) (6). As a common zoonotic disease, about 10% of human TB cases worldwide are caused by infection with *Mycobacterium bovis* (7, 8). It is of great importance for the development of animal husbandry and human health to explore the molecular pathogenesis of bTB and the evolution of the disease, to formulate better prevention and control strategies (9).

The diagnostic criteria and methods of bTB are based on the tuberculin assay and the Interferon-gamma release assay (IGRA) to detect the level of host CMI response (10). The results of the tuberculin assay mainly depend on the virulence of *M. bovis* and the immunity of the host (11), and its accuracy is usually not satisfactory (12). IGRA is a new immunodetection method for *Mycobacterium tuberculosis* infection, and the accuracy of this method is more than 95% (13). However, its high experimental technical requirements also limit its wide promotion and application (14). The two detection methods are prone to misdiagnosis of infected cattle with low CMI response levels in the early stage of infection or with low immunity in the late stage of infection (15).

In recent years, with the rapid development of molecular biology technology, the wide application of new technologies has made it possible to find more accurate and efficient methods for early diagnosis of bTB (16). The emergence of Next-generation sequencing (NGS) technology has fundamentally changed the research methods of population genetics, quantitative genetics, molecular systematics, microbial ecology, and many other research fields (17). As the leader of the new generation sequencing technology, Expression profiling by high throughput sequencing (RNA-seq) can quickly and quantitatively analyze the entire transcriptome of an organism. By performing transcriptome profiling, researchers can explore the entire gene expression network of an organism and in this way discover important genes associated with various disease phenotypes. With the rapid development of bioinformatics technology, RNA-seq has become one of the standard techniques for molecular biology research (18). Weighted correlation network analysis, also known as weighted gene co-expression network analysis (WGCNA) (19), is a widely used computer bioinformatics data mining method. It is especially suitable for the study of biological networks based on pairwise correlations between variables (20). This algorithm is based on a Scale-free network (SFN), which is a network structure closest to the pattern of the biological metabolic network. Many experimental studies have observed the scale-free network phenomenon in biological activities (21–23). As an algorithm that can be applied to most high-dimensional data, it has been widely used in genomic research. The WGCNA method is very powerful for analyzing gene expression data and can yield results and insights that are not possible in typical differential expression studies (24).

By comparing transcriptome data from bovine blood samples that infected or uninfected with *M. bovis*, the objective of this study was to elucidate the molecular functional changes and corresponding gene expression signatures in the early and late stages of bTB. To screen out early specific candidate gene markers for bTB by searching for key genes, and to find potential gene targets with diagnostic and therapeutic value.

## 2 Materials and methods

### 2.1 Public data mining

The transcriptome data used in this study were obtained from the publicly available gene database GEO (The Gene Expression Omnibus, GEO) (25). Through retrieval, the original transcriptome sequences related to this study were obtained from the dataset GSE192537 (26) for bioinformatics analysis. The original blood samples were collected from bTB-free farms, and a total of 24 blood RNA-seq sequences were collected from 12 9-month-old castrated male calves. Blood samples of 6 calves artificially infected with bTB were collected in the experimental group, and 6 healthy calves were collected in the control group. The collection time and classification information of blood samples are shown in Table 1.

**Table 1:** Sample collection time and classification information.

Sampling time	Number of blood samples in experimental group	Number of blood samples in control group	Total
8 weeks after the experiment	6	6	12
20 weeks after the experiment	6	6	12

### 2.2 Differential gene analysis

By comparing the transcriptomic gene expression information in blood samples of calves with different phenotypes, we obtained the Differential expression genes (DEGs) of the blood samples of early bTB stage with other blood samples, the blood samples of early bTB stage with the blood samples of late bTB stage with statistically significant expression. Through the Gene ontology database (GO), we explored the Biological process (BP) of bTB at each stage.

### 2.3 Analysis of WGCNA gene co-expression network

We set up the SFN network of WGCNA to search for gene sets highly associated with different stages of bTB progression. By clustering genes with similar expression patterns into gene sets with different functions. The intrinsic correlation of different gene sets and their correlation with traits were analyzed by using the hub gene, and then the correlation was sorted by weighted method. This method is a common screening method for functional gene sets in bioinformatics analysis and is widely used to identify biomarkers and drug therapeutic targets. This method can also use all gene information to search for gene sets related to traits, and avoid the problem of multiple hypothesis testing and correction due to multiple comparisons of differential genes.

The general process of analysis is as follows: Firstly, the SFN network was established and the gene set with the highest correlation with early disease cattle was identified by association analysis. BP and related biological signal pathways were enriched from the genes concentrated in the GO and KEGG databases [Kyoto Encyclopedia of Genes and Genomes (KEGG)], so that the changes in biological processes and related signal pathways that were most relevant to the disease process were identified, and to compare the differences between early and late disease cattle. Then, the Gene expression data of the genes in the gene set were used to establish a Gene interaction network (GIN), and the Hub genes in the network were found by gene network analysis. Finally, the key genes found were compared with the results of differential gene analysis to find out the significantly differentially expressed genes. The final biomarker gene set was synthesized based on all the genes with both differential expression and important roles, and their value as biomarkers was further explored.

All bioinformatics analyses were performed on R software (R Foundation for Statistical Computing, Vienna, Austria) version 4.0.2. The mainly used third-party R software packages are ClusterProfiler (27), WGCNA (28), and limma (29). Gene enrichment analysis is carried out on the website David (30), and the visualization of the gene interaction network is shown by the open-source software Cytoscape (31). In all statistical tests, the difference was considered significant by bilateral test *p* < 0.05, and in analyses involving multiple comparisons, Fisher exact test FDR < 0.05 was used to test the *p*-value.

### 2.4 Genetic marker validation based on random forest method

Random forest (RF) is a commonly used machine learning method. Its essence is a classifier containing multiple decision trees, which can predict the input samples through parameter training. In this study, the mRNA expression level of the selected key genes was used as the independent variable, the status of normal and disease was set as the dependent variable 1, and the blood samples of early bTB stage and other blood samples were set as the binary dependent variable 2.

Firstly, the genes that are more important for classification are screened by the method of stochastic forest forward screening. Then, these more important genes were used to establish the Logistic regression (LR) diagnostic model, and internal cross-validation was used to verify whether the pivot genes could be used as disease-related genetic markers. The Receiver operating characteristic curve (ROC) was used to evaluate the disease diagnosis model established by genetic markers, and the Area under the curve (AUC) was calculated. This section mainly operates on Python version 3.8.0, using third-party software sklearn (32).

## 3 Results

### 3.1 Transcriptional phenotypic heterogeneity analysis

**Figure 1:**
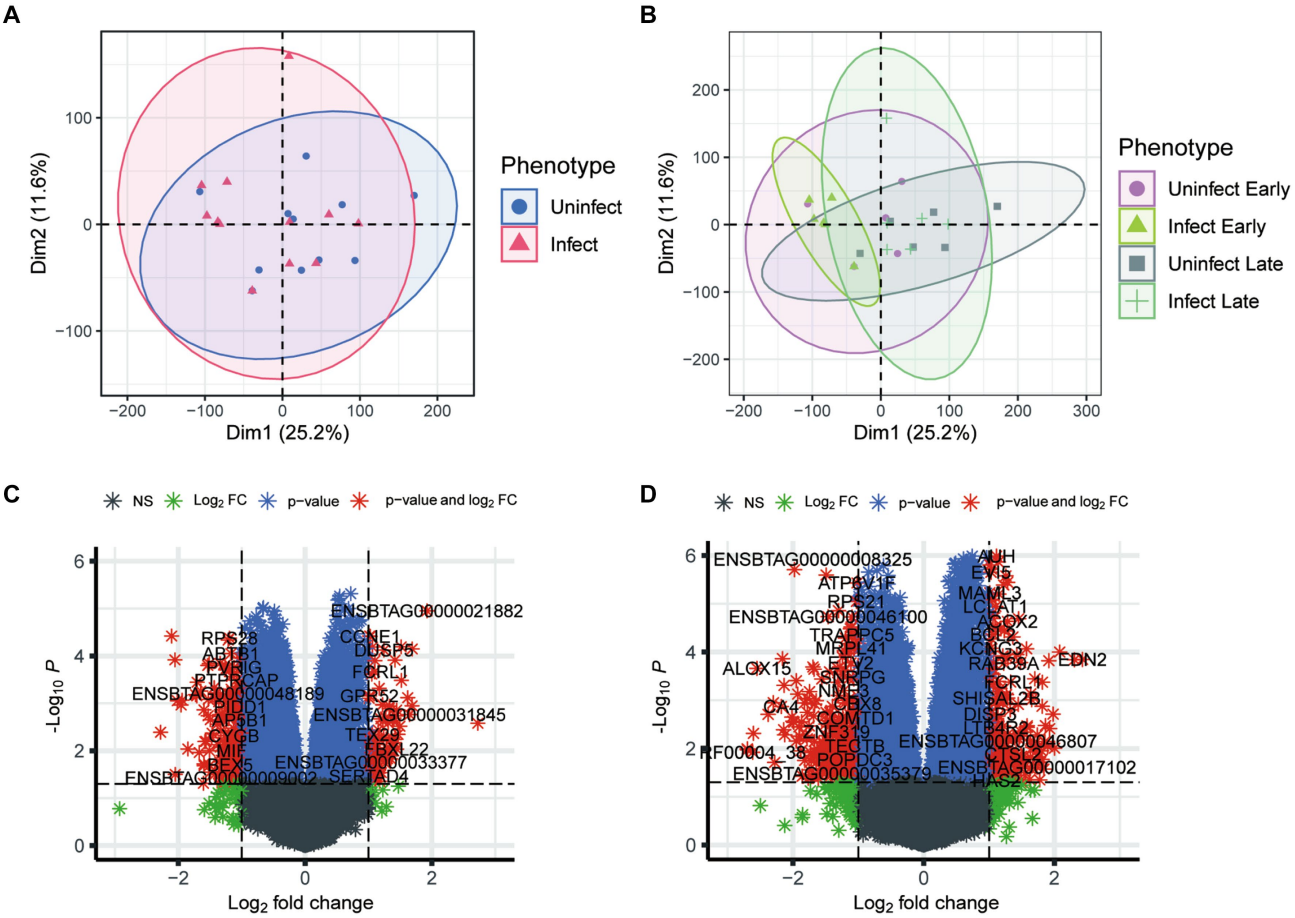
Analysis of transcriptional phenotypic heterogeneity in bovine tuberculosis blood samples. **(A)** PCA analysis of gene expression in infected and uninfected groups. **(B)** PCA analysis of gene expression in infected and uninfected group at different periods. **(C)** Volcano plot shows the differential expression genes between the blood samples of early infection and other blood samples. **(D)** Volcano plot shows the differential expression genes between the blood samples of early infection and those of late infection blood samples.

To investigate the effects of TB at the transcriptional level, we utilized principal component (PCA) analysis to visualize the heterogeneity of gene expression among the phenotypes. As shown in Figure 1A, few discrepancy was observed between infected and non-infected groups. Furthermore, in the early groups, bTB had a limited impact on gene regulation. However, the discrepancy between infected and non-infected groups enhanced in the late phenotypes (Figure 1B). These results suggest that the development of TB could be occult and the impact of TB on gene expression was gradually enhanced with the progression of TB.

After batch correction, a total of 1,600 differential genes were obtained from the blood samples of early bTB stage and other blood samples, among which 811 differential genes were up-regulated and 789 were down-regulated (Figure 1C). The top 10 up-regulated (and down-regulated) DEGs were detailed in Supplementary Table S1. GO enrichment analysis of the main biological process BP was performed for the up-regulated and down-regulated genes, respectively. After batch correction, a total of 3,308 differential genes were obtained from the early and blood samples of late bTB stage, among which 1,476 differential genes were up-regulated and 1832 were down-regulated (Figure 1D). The top 10 up-regulated (and down-regulated) DEGs were detailed in Supplementary Table S2. GO enrichment analysis of the main biological processes of BP was performed for the up-regulated and down-regulated genes, respectively. The top 10 enriched gene sets of up-regulated genes are shown on the left and the top 10 enriched gene sets of down-regulated genes are shown on the right. In the comparison of early and the blood samples of late bTB stage, late bTB stage had more enrichment of biological processes related to protein and synthesis, which reflected the extensive pathological manifestations of tuberculosis granuloma formation in late bTB stage.

### 3.2 Analysis of WGCNA weighted gene coexpression network

#### 3.2.1 Clustering of coexpressed genes

All genes with similar expression patterns were clustered into large gene modules in the form of decision trees, and the results of the dendrogram were cut using the dynamic clipping method. Finally, a total of 66 large gene modules consisting of genes with similar expression were obtained. Supplementary Figure S1A presents the results of all modules and their decision tree clustering. To further test the clustering results, 400 genes (Supplementary Table S3) were randomly selected from the network to sketch the clustering heat map. Supplementary Figure S1B showed that the clustering results of the heat map, and genes classified as the same module were clustered together.

#### 3.2.2 Explore gene expression modules associated with early bovine tuberculosis

To explore the differences and similarities of gene expression patterns in blood samples, all experimental cattle samples were divided into four phenotypes: early-infected cattles (8 weeks), healthy cattles (8 weeks), late-infected cattles (20 weeks), and healthy cattles (20 weeks). Correlation analysis was made between similar gene expression modules identified previously and phenotypes to search for the gene expression modules with the highest correlation with the blood samples of early infected cattle. The clustering results are shown in Supplementary Figure S1C. The brown and yellow-green gene modules were related to the phenotype of early infected cattle.

**Figure 2:**
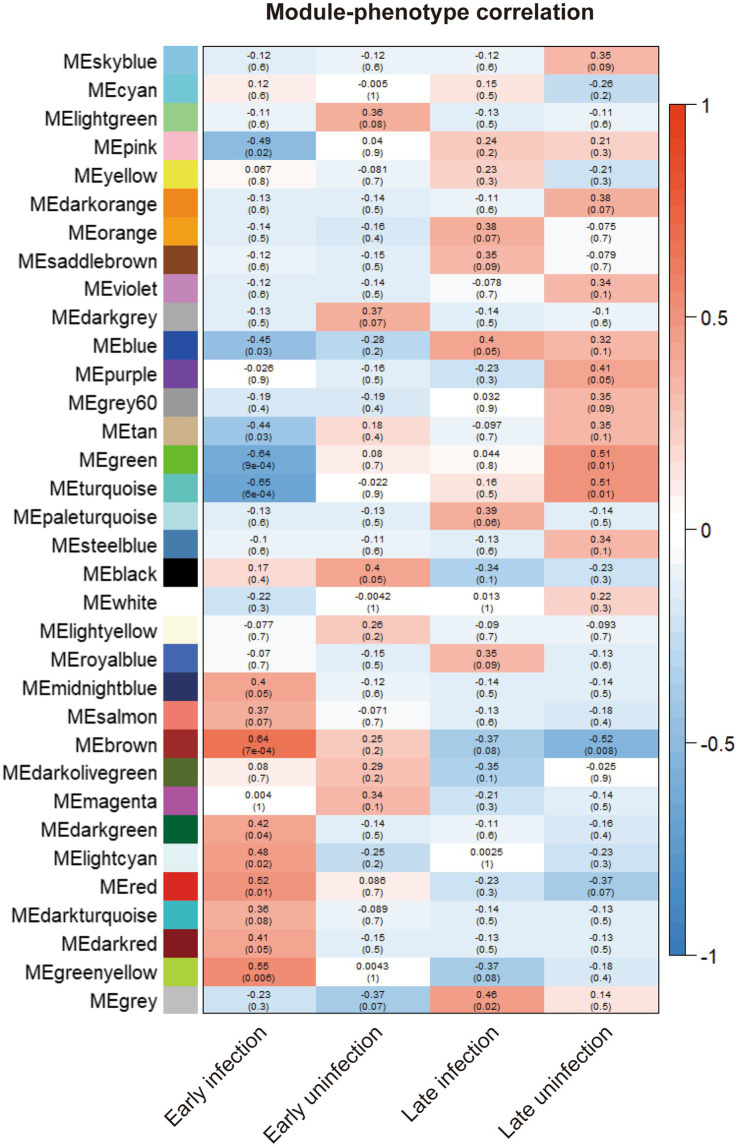
The correlation between the gene modules and all phenotypes. The correlation indexes are labeled in each cell of the heatmap and the *p*-values are labeled in the brackets below.

To statistically test the clustering results and compare the correlation of different gene expression modules with all phenotypes, Spearman correlation analysis was performed for all gene expression modules with all phenotypes. The results of all correlation analyses are shown in Figure 2. Both the brown module and the yellow-green module had a high correlation with the phenotype of early infected bovine blood samples, and the correlation was 0.64 and 0.55, respectively, and both were statistically significant. The genes in the two gene modules were extracted, resulting in 1923 genes in the brown gene expression module and 236 genes in the yellow-green gene expression module.

#### 3.2.3 To explore the correlation between genes of specific gene expression modules and the early phenotype of bovine tuberculosis

**Figure 3:**
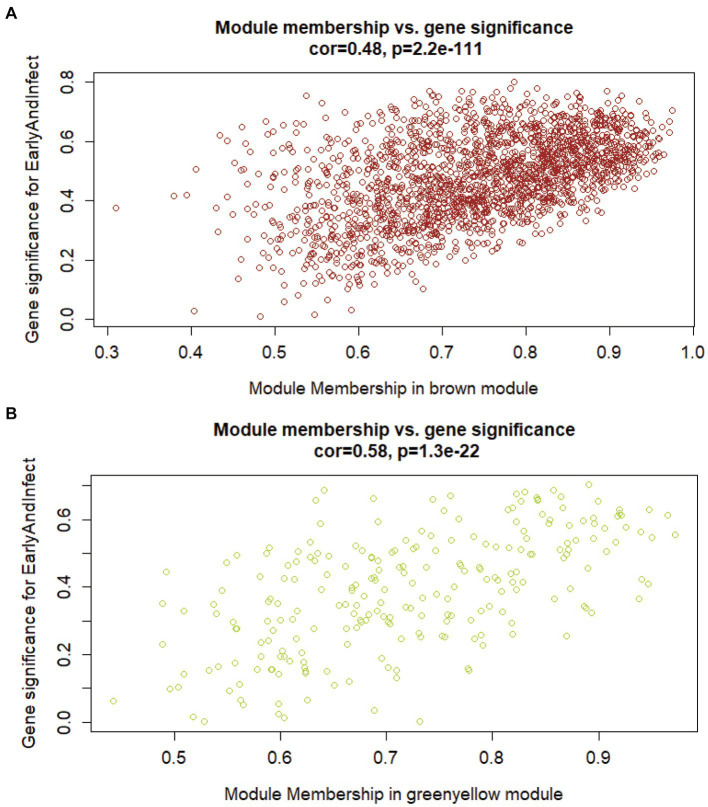
The correlation between genes in modules of interest and the early infection phenotype. **(A)** Correlation between brown gene expression module and early infection phenotype. **(B)** Correlation between yellow-green gene expression modules and early infection phenotype.

To search for the genes most associated with the phenotype, it is necessary to compare the correlation between the genes in the above two modules and the phenotype. Firstly, the correlation matrix between the two gene expression modules and the genes in the modules should be calculated respectively, and then the module genes should be calculated. Finally, the two correlation matrices are superimposed to evaluate the correlation between genes and phenotypes in the module. Figure 3 shows the correlation and significance of genes and phenotype in the brown and yellow-green modules, respectively. The correlation between the two modules and the early infection phenotype was 0.48 and 0.58. The expression of genes in the yellow-green module had a higher correlation with the early infection phenotype. The important genes with a high correlation with phenotype in the yellow-green module have a higher value as early biomarkers of bTB.

#### 3.2.4 Gene interactions in specific gene expression modules and their biological functions

The interaction information between genes in the yellow-green gene expression module was exported and the gene interaction network was established. Supplementary Figure S2 illustrates the interaction network of genes in the yellow-green gene expression module, most of these genes are closely interconnected and may be involved in the regulation of specific biological processes together (Supplementary Table S5).

**Figure 4:**
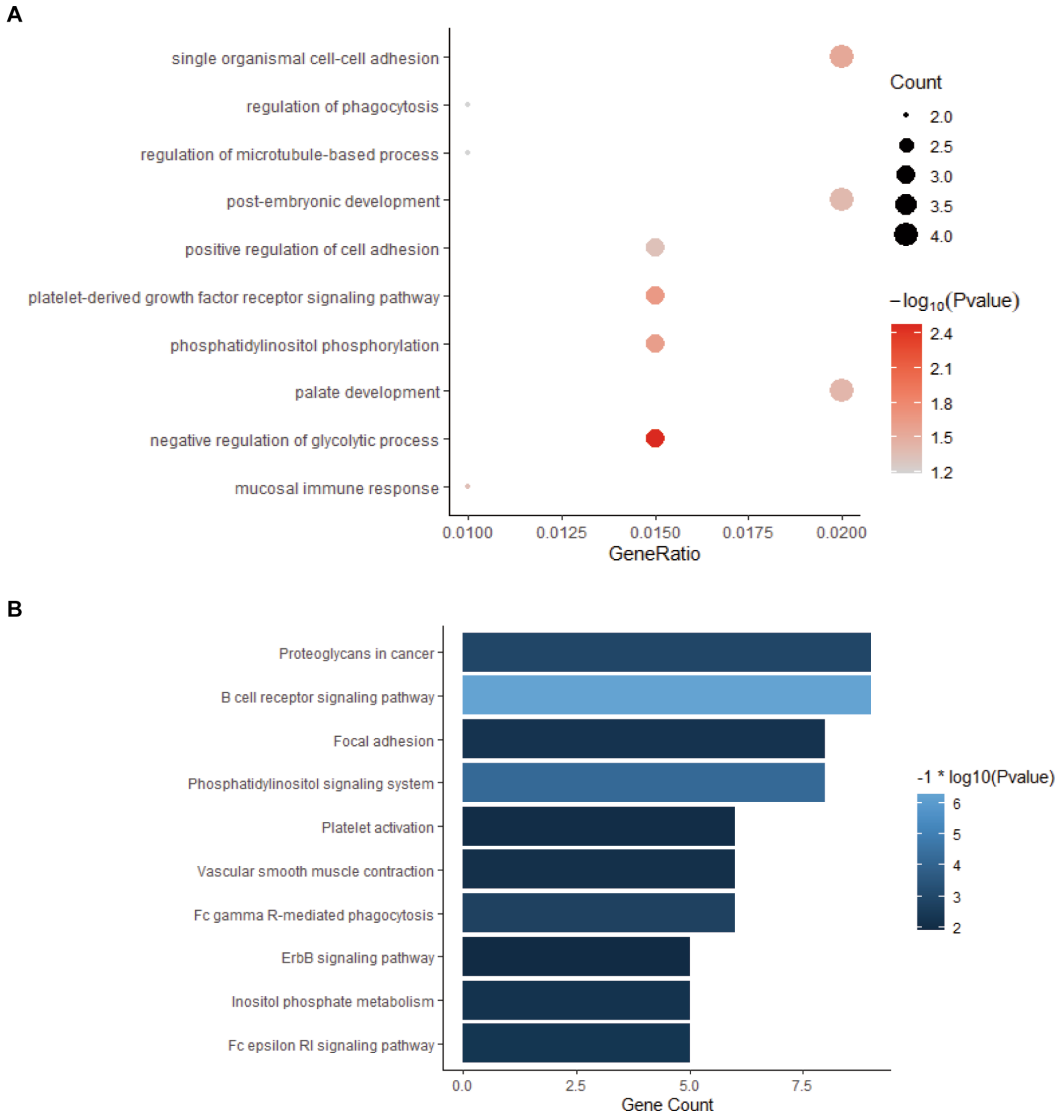
GO and KEGG enrichment analysis of the genes in yellow-green module. **(A)** GO enrichment analysis of genes in yellow-green module. **(B)** KEGG enrichment analysis of genes in yellow-green module.

All the genes in the yellow-green gene expression module were enriched by GO and KEGG analysis. The results of GO and KEGG enrichment analysis are shown in Figure 4. The genes in this gene expression module were mainly enriched in biological functions and signaling pathways such as cell adhesion, immune activation receptors, and antigen presentation receptors, especially related to B cell-related antigen presentation system. These results indicated that there was significant immune activation and recruitment of immune cells in the early stage of bTB.

#### 3.2.5 The key gene of gene interaction network

Hub genes are also known as key genes, which are similar to housekeeping genes. They play an irreplaceable role in regulating specific biological processes and can have a major impact on specific biological processes through the expression of many genes in their expression regulatory network.

**Figure 5:**
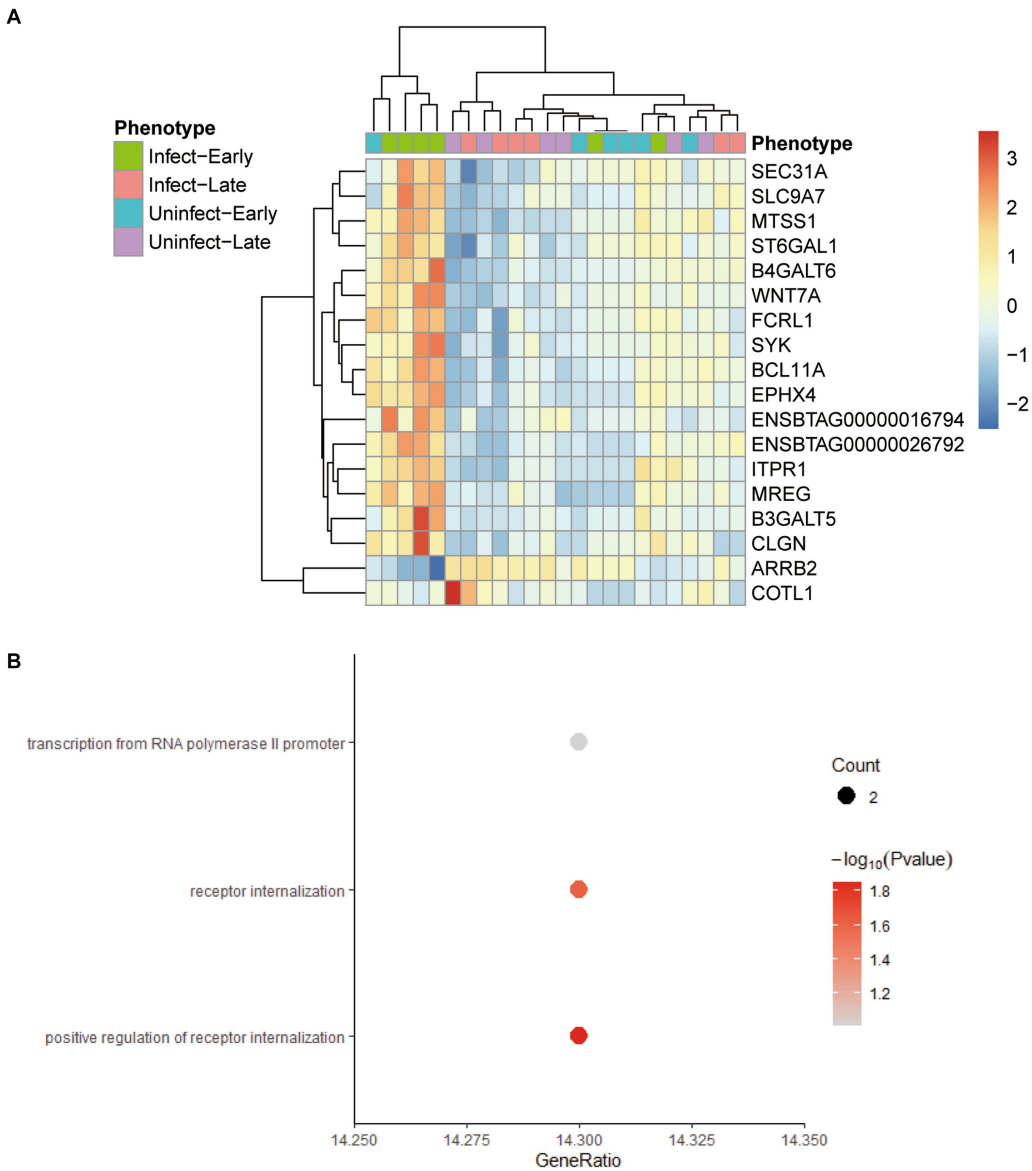
The characteristics of 18 hub genes in the yellow-green module. **(A)** Heatmap plot shows the relative expression of the 18 hub genes across the different phenotypes. **(B)** The GO enrichment analysis of the 18 hub genes.

The screening criteria for hub genes were as follows: (1) the correlation between the gene and the module >0.2; (2) The gene was directly or indirectly related to 80% of the genes in the module; (3) The statistical test of the correlation between the gene and the module was <0.05. We found 18 hub genes in the yellow-green module: ARRB2, B3GALT5, B4GALT6, BCL11A, CLGN, COBLL1, ENSBTAG00000016794, ENSBTAG00000026792, EPHX4, FCRL1, ITPR1, MREG, MTSS1, SEC31A, SLC9A7, ST6GAL1, SYK, WNT7A. Comparing these genes with the differential genes found in 3.1, it was found that these 18 genes were the differential genes between the blood samples of early bTB stage and all other blood samples, and had the potential to be biomarkers (Figure 5A). Figure 5B shows the GO enrichment analysis of these 18 genes, which are found to be mainly related to receptor regulation.

### 3.3 Genetic marker validation based on random forest method

#### 3.3.1 Random forest screening of important genes

**Figure 6:**
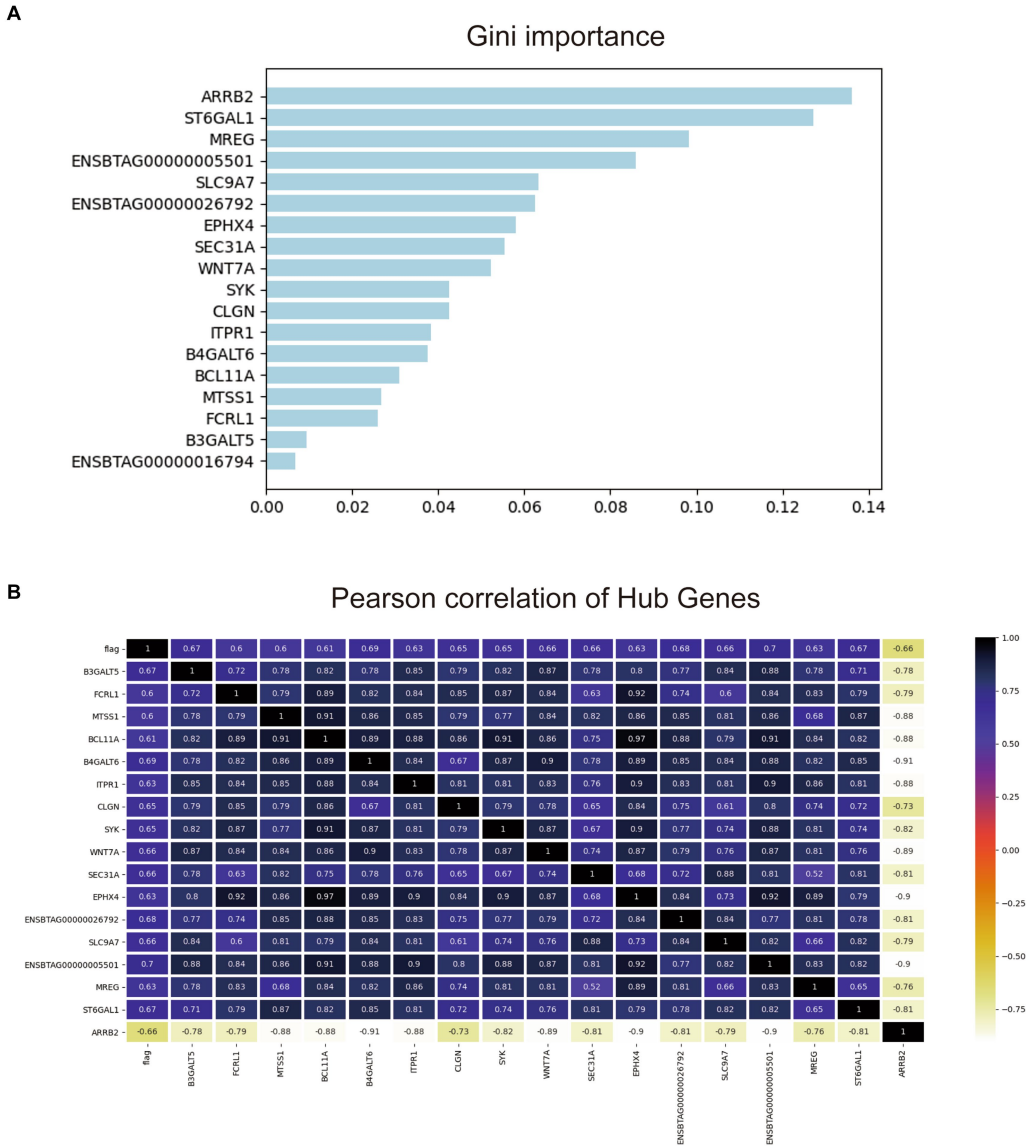
Gini important ranking and the correlation matrix of the 18 hub genes. **(A)** Gini important ranking of the genes for the random forest diagnostic model. **(B)** The correlation matrix of the 18 hub genes and the prediction.

To further screen the most valuable key genes for the diagnosis of early-stage bTB, the transcriptome expression matrix of these 18 genes was extracted from the original gene expression matrix and then put into the random forest model. One hundred classifiers were randomly built and the contribution of each gene to the classification was calculated. Figure 6A illustrates the ranking of the contribution of these 18 genes to the diagnostic classification.

As shown, Figure 6B shows that it is evident that almost all of these 18 genes have moderate to strong correlations with taxonomic phenotypes. Among them, 14 genes had a strong correlation with each other with Pearson coefficient > 0.8. The other four genes were ENSBTAG0000001679, B3GALT5, FCRL1, and MTSS1. Since the total number of experimental samples N was 24, the number of predictors ≈N/10 was required to avoid overfitting or underfitting the established model. Therefore, the contribution of the remaining four genes to the diagnostic model was compared one by one by using random forest forward screening. The predictors of FCRL1 and MTSS1 were screened to establish Logistic regression diagnostic model. The correlation of the two genes with the four phenotypes is shown in Supplementary Figure S3.

#### 3.3.2 Establishment and verification of early diagnosis model of bovine tuberculosis

**Figure 7:**
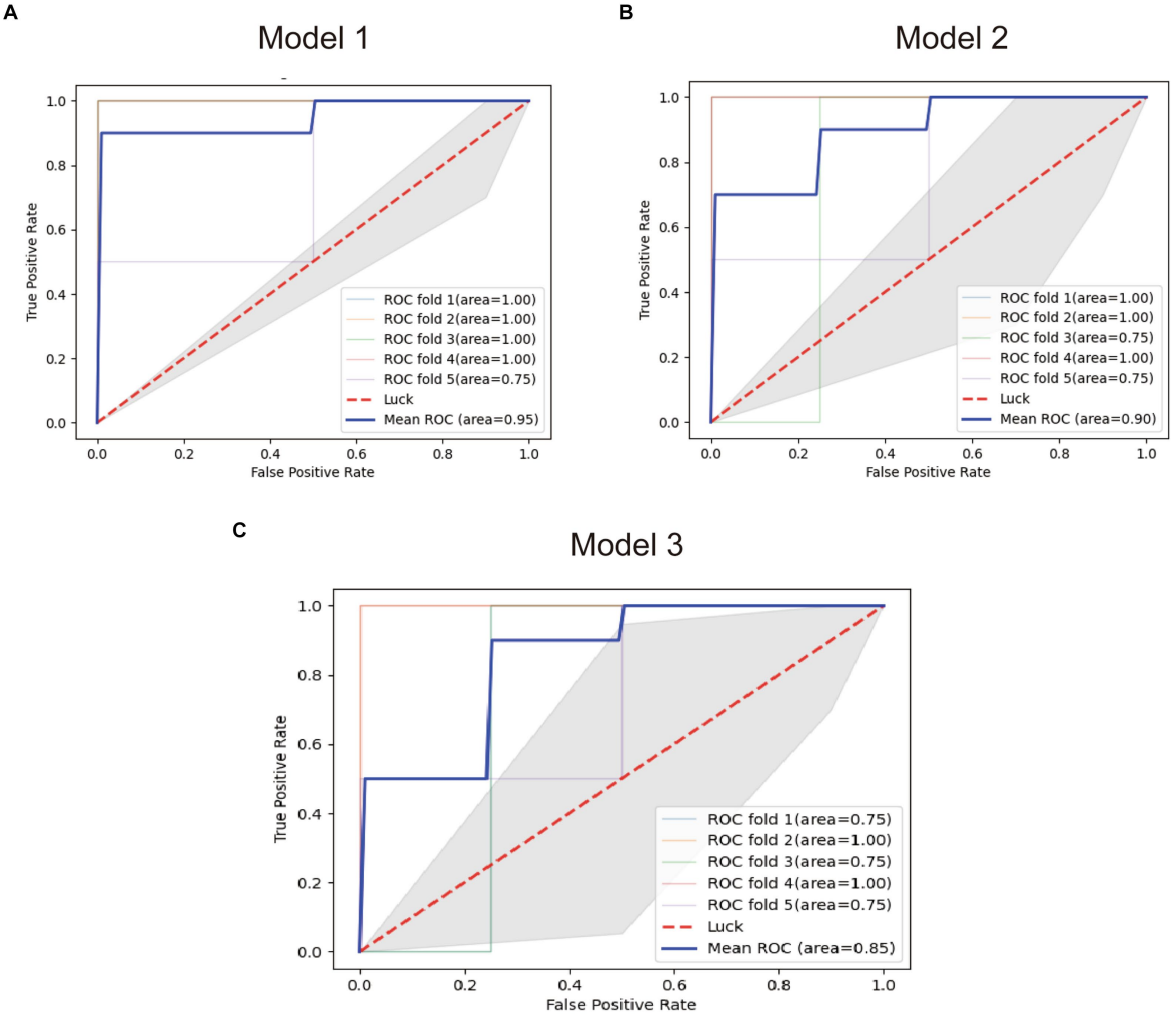
ROC plots of the diagnostic models. **(A)** ROC plot of the model 1. **(B)** ROC plot of the model 2. **(C)** ROC plot of the model 3.

FCRL1, MTSS1, and the combined model were used to establish diagnostic models (Model 1, Model 2, and Model 3), and the performance of the models was tested by the in-sample five-fold cross-validation method. The average AUC obtained from the five tests was used as the final evaluation standard to compare the performance of the three models. Figure 7 presents the test results of the three models.

The results showed that the diagnostic Model constructed with FCRL1 as a predictor had the best performance with an average AUC of 95%, while the diagnostic model constructed with two genes had the worst performance with an average AUC of 85%. The Pearson correlation coefficient between the two genes is 0.79, which is very close to the screening threshold of 0.8. Therefore, the model formed by the two genes may have multicollinearity and lead to poor fit. However, the average AUC of Model 2 constructed with MTSS1 as a predictor was 85%, and its diagnostic efficacy was lower than that of Model 1. Therefore, FCRL1 as a mRNA marker of bTB in blood samples of early bTB is ideal.

## 4 Discussion

Bovine TB is a significant health problem for both animals and humans, with surveys showing that 85% of cattle and 82% of humans live in areas where the disease is endemic (33). According to another study, it is conservatively estimated that about 2% of human pulmonary TB infections and 8% of extrapulmonary TB cases are caused by bTB transmission (34). TB is a zoonotic disease that deserves extensive attention.

Youngstock is vulnerable to bTB (35). Calves are also the classic host model used for bTB vaccine development (36, 37). Previous studies have revealed that bTB was associated with classic immune regulatory networks, such as the STRING pathway and CTSG network (38, 39). In addition to the immune regulatory network, some immune proteins were also identified as the characteristic transcriptome alterations of bTB, such as the CCL8 (40), CCR5 (41), CXCL10 (42), IP-10 (43), and CXCL9 (44). Although several biomarkers have been developed previously for bTB diagnosis, these transcriptome alterations show limited value in identifying the early infection (45–47).

In this study, we performed a multi-dimensional analysis of the transcriptome data of bovine whole blood by mRNA-Seq technology, aiming to clarify the changes in gene expression profiles between the early stage (8 weeks) and the late stage (20 weeks) of bTB by bioinformatics technology. To find mRNA markers in the blood samples of early bTB stage with practical value by analyzing the changes in gene expression profiles, to diagnose early bTB more accurately and reduce the economic losses and health damage caused by the disease.

By analyzing the transcriptome data of whole blood samples from different periods and different phenotypes (normal and infected samples), this study elucidated the characteristic biological processes of blood samples with different infection phenotypes in different periods. Through differential gene analysis, we found that immune-related biological processes seemed to be more active in the blood samples of early bTB stage than other samples and immune-related molecules in the blood samples had the potential to be used as diagnostic markers for early bTB infection. By comparing the transcriptomes of the early and late stages of the disease, we found that there were more biological processes related to protein synthesis in the late stage than in the early stage, which confirmed the pathophysiological changes of the formation of a large number of encapsulated granulomas and a large number of protein deposits in the lesions in the late stage of tuberculosis from the aspect of gene regulation.

After establishing the WGCNA-weighted gene co-expression network, we successfully identified 66 gene expression modules with different biological process regulation functions by expression pattern similarity. By analyzing their correlation with different phenotypes, we found the gene expression modules significantly associated with this phenotype in the early stage of bTB. The module is composed of 236 genes whose expression is highly correlated, confirming that they may be jointly involved in the regulation of specific biological processes. Through GO and KEGG enrichment analysis, we found that the genes in this module were mainly involved in the regulation of B cell-related antigen presentation system, which confirmed that there was an obvious biological process of immune activation and immune cell recruitment in the blood samples of early bTB stage, and it was also found that this process was mainly completed by the regulation of immune receptors. Gene interaction networks show that, There are 18 genes in this module associated with early bTB, most of which function as hub genes related to the regulation of immune receptors (ARRB2, B3GALT5, B4GALT6, BCL11A, CLGN, COBLL1, ENSBTAG00000016794, ENSBTAG00000026792, EPHX4, FCRL1, ITPR1, MREG, MTSS1, SEC31A, SLC9A7, ST6GAL1, SYK, WNT7A). Through correlation analysis, we found that the expression changes of these genes were moderately correlated with this phenotype at the early stage of bTB, suggesting that these genes are gene targets with diagnostic value and potential value for disease treatment. Through the random forest screening factor and LR regression model, we established three models for the diagnosis of early bTB. These models all showed excellent diagnostic performance, indicating that blood transcriptome examination has a certain diagnostic value. By comparing the performance of the models, we found that the diagnostic model composed of FCRL1 was the best.

FCRL1, full of Fc receptor-like 1, is located on chromosome 3 of cattle and is widely present in the mammalian genome. It is specifically expressed on the surface of the cell membrane of B lymphocytes and is involved in the regulation of immune activation and immune function of B cells (48, 49). Different species express different Fc receptors on B cell membranes. Human B cells express FCRL1 to FCRL5 on the plasma membrane, while mouse B cells only express FCRL1 and FCRL5 (50). Quantitative reverse transcription polymerase chain (RT-PCR) showed that the expression of FCRL1 began in pre-B cells, and then was significantly increased in ordinary B cells and memory B cells (51). *In vitro* studies have shown that antibody-mediated cross-linking of the extracellular domain of FCRL1 leads to intracellular tyrosine residues phosphorylation, enhances the binding ability of B cells to antigens, and induces Ca2+ mobilization and proliferation of B cells, confirming that FCRL1 is an important regulator of B cell activation (52, 53). Another study showed that FCRL1 also plays a positive regulatory role in T-cell-dependent and independent antibody responses (48). These findings confirm that FCRL1 is an important factor in mammalian immune regulation. In this study, the mean ROC of the diagnostic model established by FCRL1 reached 95%, indicating that FCRL1 can be used as an mRNA biomarker in the blood samples of early bTB stage.

Compared to the previously published study (25), this study explored the association between gene expression module and bTB depending on a WGCNA-based analysis instead of the simple DEG analysis and GO analysis. Through correlation analysis, we identified the most relevant gene regulatory network of early bTB infection. Rather than providing an equivocal gene list, we obtained a promising biomarker to screen the early bTB infection through a validated machine learning algorithm.

However, this study was a single-center study with a short study time and small sample size. In this study, we did not consider the expression profiles of other infectious diseases. The gene marker FCRL1 screened in this study is a widely distributed immunoregulatory gene, and its specific association with bTB still needs to be confirmed by large-scale epidemiological studies and basic experiments. Therefore, the diagnostic model established in this study has application value only when other infectious diseases are excluded, which limits its popularization value to a certain extent. As a key zoonosis, it is still a challenge to diagnose bTB accurately in the early stage. At the same time, it is conducive to the development of the livestock economy and the protection of human life and health safety to update the definition based on evidence-based medicine, prevent and manage disease and risk control, and conduct related large-scale clinical trials.

## 5 Conclusion

In this study, we screened candidate mRNA marker genes with specificity in blood samples of early bTB stage by transcriptome analysis. The results showed the transcription activity of genes involved in immune-related biological processes was more active in the early stage of bTB than in the late stage. There were obvious immune activation and recruitment of immune cells in the early stage of bTB. Changes in immune receptor regulation have been linked to the pathophysiological processes in the early stages of bTB. FCRL1 has the potential to be an mRNA biomarker in the blood samples of early bTB stage. The results of this study provide support for the early diagnosis of bTB.

## Data availability statement

Publicly available datasets were analyzed in this study. This data can be found here: https://www.ncbi.nlm.nih.gov/geo/; GSE192537.

## Ethics statement

Ethical approval was not required for the studies on animals in accordance with the local legislation and institutional requirements because only commercially available established cell lines were used.

## Author contributions

KN: Methodology, Resources, Writing – original draft. DJ: Investigation, Software, Writing – original draft, Writing – review & editing. XS: Data curation, Formal analysis, Software, Writing – original draft. LY: Formal analysis, Project administration, Supervision, Writing – review & editing. LZ: Conceptualization, Data curation, Methodology, Writing – original draft. HN: Conceptualization, Funding acquisition, Supervision, Validation, Writing – original draft.
