# Supplementary material for: Screening of mRNA markers in early bovine tuberculosis blood samples
Source: Front Vet Sci. 2024 Apr 5;11:1330693. doi: 10.3389/fvets.2024.1330693 (PMC11026862; doi:10.3389/fvets.2024.1330693)
Supplement: Supplementary file 1 [file Image_1.pdf]

Supplementary Material

1 Supplementary Figures and Tables

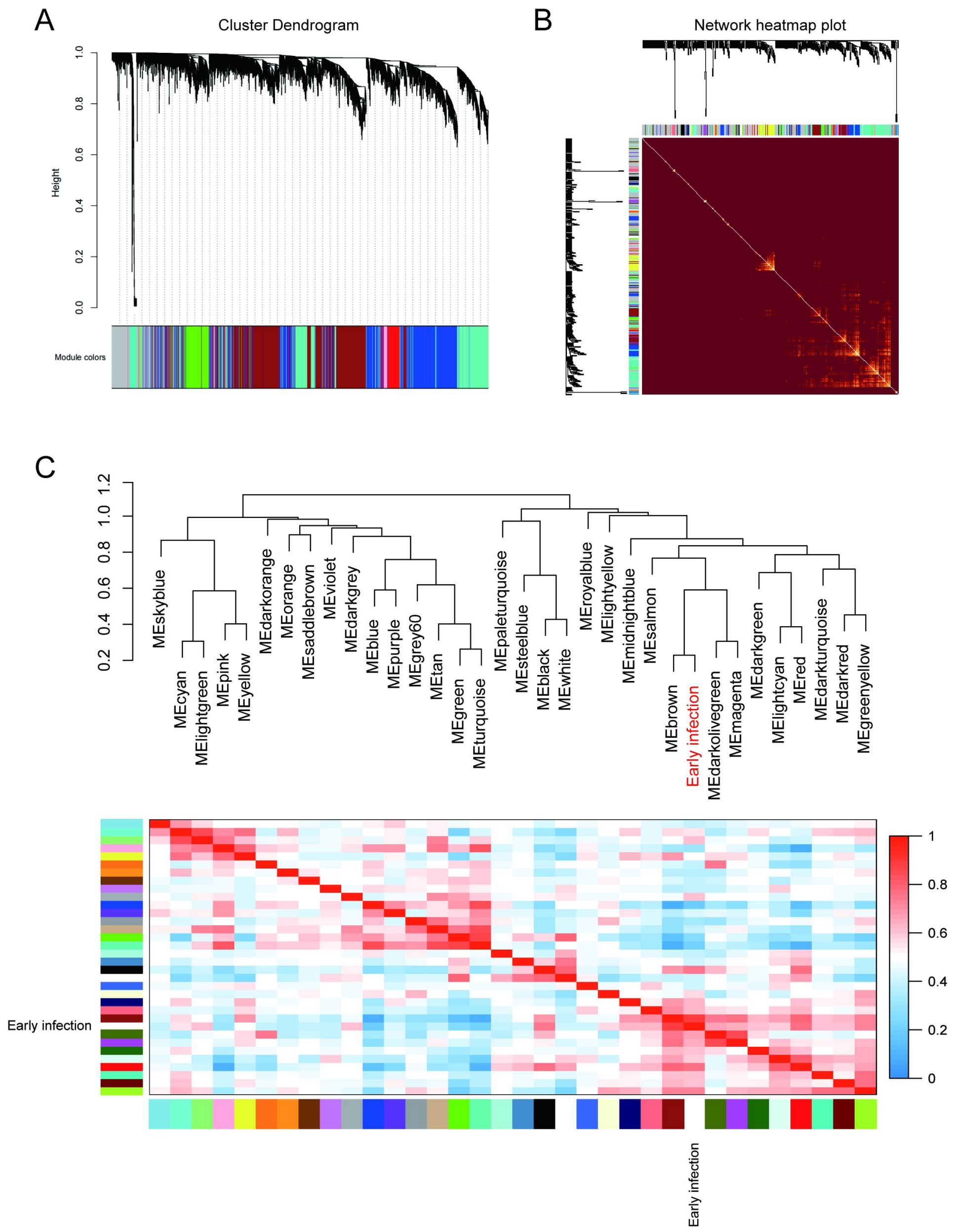

Supplementary Figure 1. Analysis of weighted gene coexpression network. (A) Decision tree clustering for identification of gene modules. (B) The heatmap of correlation matrix of the 400 random-selected genes. (C) Correlation clustering among gene modules and the phenotype of early-infected blood samples.

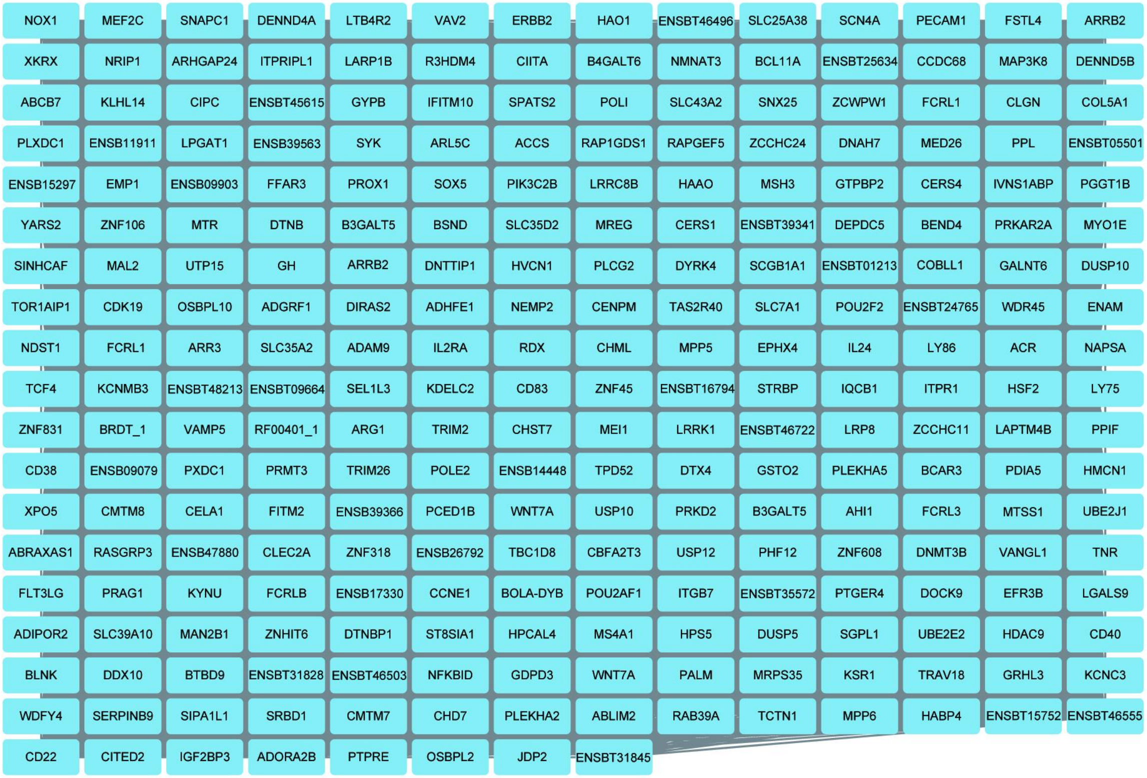

Supplementary Figure 2. Gene interaction network of genes in the yellow-green module.

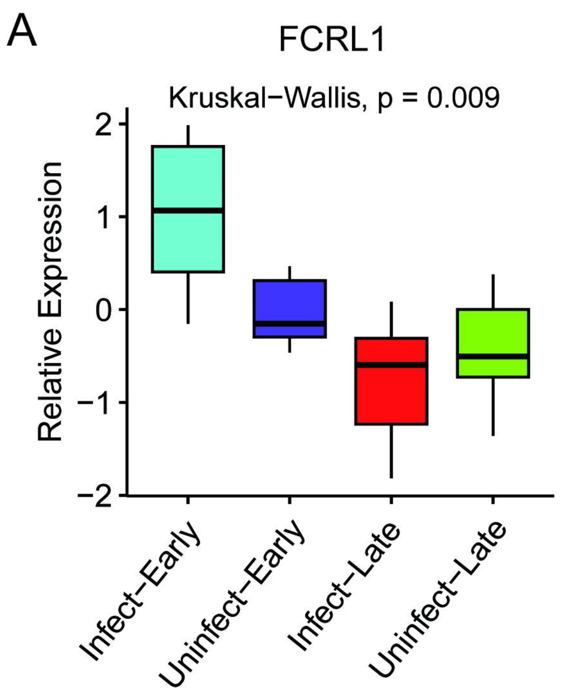

Supplementary Figure 3. The relative expression of FCRL1 and MTSS1 across all phenotypes. (A) The relative expression of FCRL1 across all phenotypes. (B) The relative expression of MTSS1 across all phenotyp
